# Conformer-Specific Photoelectron Spectroscopy of Carbonic Acid: H_2_CO_3_

**DOI:** 10.1021/acs.jpclett.4c00343

**Published:** 2024-03-01

**Authors:** Keisuke Kanayama, Hisashi Nakamura, Kaoru Maruta, Andras Bodi, Patrick Hemberger

**Affiliations:** † Laboratory for Synchrotron Radiation and Femtochemistry, Paul Scherrer Institute, CH-5232 Villigen PSI, Switzerland; ‡ Institute of Fluid Science, Tohoku University 2-1-1 Katahira, Aoba, Sendai, Miyagi 980-8577, Japan; § Graduate School of Engineering, Tohoku University, 6-6 Aramaki Aza Aoba, Aoba, Sendai, Miyagi 980-8579, Japan

## Abstract

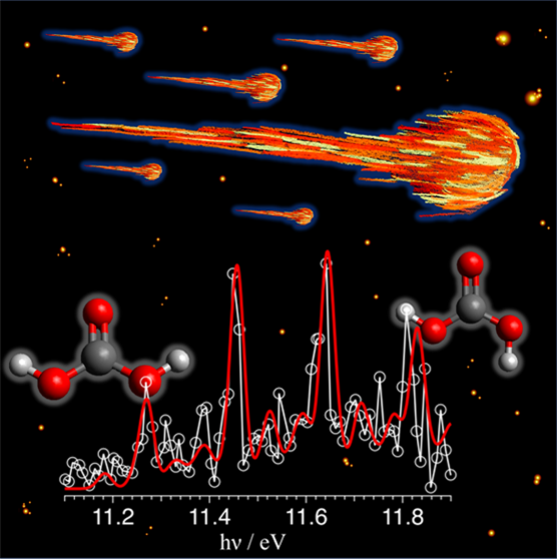

Carbonic acid (H_2_CO_3_) is a fundamental species in biological, ecological, and astronomical systems. However, its spectroscopic characterization is incomplete because of its reactive nature. The photoionization (PI) and the photoion mass-selected threshold photoelectron (ms-TPE) spectra of H_2_CO_3_ were obtained by utilizing vacuum ultraviolet (VUV) synchrotron radiation and double imaging photoelectron photoion coincidence spectroscopy. Two carbonic acid conformers, namely, *cis–cis* and *cis–trans*, were identified. Experimental adiabatic ionization energies (AIEs) of *cis–cis* and *cis–trans* H_2_CO_3_ were determined to be 11.27 ± 0.02 and 11.18 ± 0.03 eV, and the cation enthalpies of formation could be derived as Δ_f_*H*°_0K_ = 485 ± 2 and 482 ± 3 kJ mol^–1^, respectively. The *cis–cis* conformer shows intense peaks in the ms-TPES that are assigned to the C=O/C–OH stretching mode, while the *cis–trans* conformer exhibits a long progression to which two C=O/C–OH stretching modes contribute. The TPE spectra allow for the sensitive and conformer-selective detection of carbonic acid in terrestrial experiments to better understand astrochemical reactions.

Carbonic acid (H_2_CO_3_, **1**) plays a fundamental role in our daily lives as constituent of the carbonate buffer stabilizing the pH of blood^1^ and as a contributor to ocean acidification.^2,3^ Laboratory experiments have been carried out on the production and spectroscopic characterization of **1** in the condensed phase to investigate its astrochemical role in water-rich ices when abundant CO_2_ and CO are present.^4−13^ Gaseous carbonic acid, **1**, a long predicted component in extraterrestrial environments,^14−16^ has only recently been detected toward the Galactic Center molecular cloud G+0.693-0.027 by Yebes and IRAM radio telescope measurements.^17^ The chemistry of carbonic acid formation in the interstellar medium (ISM) is suggested to proceed at low temperatures along radical-driven routes on icy dust-grain surfaces yielding *cis* and *trans* HOCO radicals as intermediates, which form **1** upon reaction with a second hydroxyl radical.^9^ An electron-driven route followed by radical association reactions to form **1** was also suggested.^16^ These pathways may well be active in comets, such as Hale–Bopp, on the icy surfaces of the Galilean moons Europa and Callisto, as well as on Mars.^18,19^ Based on computational and IR spectroscopic studies,^20−23^**1** appears in three conformers with a relative stability of *cis–cis* (**1cc**) > *cis–trans* (**1ct**) ≫ *trans–trans* (**1tt**), as determined by intramolecular H-bonding and shown in Scheme 1. The formation of **1cc** is further promoted by H atom tunneling in **1ct**, as shown by Wagner et al.,^24^ which makes **1cc** likely the most abundant conformer in the ISM. Indeed, Sanz-Novo et al. estimated the **1cc** to **1ct** ratio to be 25:1; however, they could only detect **1ct** by radio astronomy because of the low dipole moment of **1cc**.^17^

**Scheme 1:**
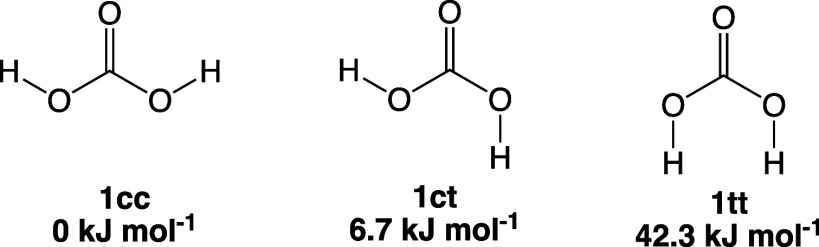
Three Conformers of Carbonic Acid (**1**) Relative energies taken from Reisenauer et al.^23^

The spectroscopic characterization of gas-phase **1** is incomplete and limited to the infrared (IR) and microwave ranges. **1** was first captured by Terlouw et al.^25^ by heating ammonium bicarbonate (NH_4_HCO_3_) and using electron ionization mass spectrometry for detection. Mori et al.^22,26^ produced **1** using a pulsed discharge nozzle with a CO_2_/Ar/H_2_O and measured the microwave spectrum of both **1cc** and **1ct**. Bernard et al.^27,28^ investigated the IR spectra of **1** in a low temperature Ar matrix produced through protonation of HCO_3_^–^ with HCl in a methanolic solution or with HBr in an aqueous solution.^5,29^ In a series of experiments the α- and β-polymorphs of solid **1** were discussed.^5,18,27^ This assignment was later corrected by the matrix isolation IR spectrum of Reisenauer et al.,^23^ who produced **1** via 2-fold isobutene (C_4_H_8_) loss from di-*tert*-butyl carbonate (DTBC, **2**) by vacuum flash pyrolysis.^30^ They found that the methyl ester of carbonic acid caused the feature in the IR spectrum, incorrectly assigned to the α-polymorph.^30^

Multiplexed photoionization^31,32^ and photoelectron spectroscopic^33−36^ methods offer high sensitivity (<1 ppb),^37^ multiplicity (detection of various species at the same time), and high selectivity because constitutional isomers and diastereomers including conformers often have different ionization energies (AIEs) and vibronic structures. Thus, photoionization tools are ideally suited as a detection tool in terrestrial astrochemistry experiments to sample from complex reactive environments and probe the entire chemistry at conditions relevant to the interstellar medium. Photoelectron photoion coincidence (PEPICO) spectroscopy^38−40^ combines mass spectrometry and photoelectron spectroscopy to record photoion mass-selected threshold photoelectron spectra (ms-TPES). This allows for an isomer-selective assignment^38^ of reactive intermediates in catalysis,^39^ combustion,^41^ and in reactions modeling the ISM, which is a clear advantage compared to conventional photoelectron spectroscopy done in laboratories, suffering from spectral congestion due to the overlap of PE bands from different molecules.^33^ This motivated us to investigate **1** using vacuum ultraviolet (VUV) synchrotron radiation to obtain the threshold photoelectron spectrum, to determine cation energetics and the geometry change upon ionization as driven by the electronic structure of **1**, similar to the approach employed for the picolyl radicals and *m*-xylylene or *m*-benzyne diradicals.^42−44^ This study will enable laboratory detection of **1** in complex reaction mixtures by means of photoionization and photoelectron spectroscopy, which is more sensitive as compared to conventional absorption methods (e.g., rotational spectroscopy).^45^ Furthermore, the ionization energies and the threshold photoelectron spectrum measured in this work are important properties to develop strategies to explore the excited states by resonance-enhanced multiphoton ionization (REMPI) and time-resolved photoelectron spectroscopy.

Carbonic acid (**1**) was produced by flash pyrolysis of the DTBC (**2**) precursor diluted in helium or argon following Reisenauer’s strategy.^23^ See the Supporting Information (SI) for other strategies^25,46,47^ to produce **1**, which were also attempted but failed in this study. The pyrolysis products expanded into high vacuum and formed a molecular beam. The sample was ionized by VUV synchrotron radiation and detected by PEPICO spectroscopy. Mass spectra at 11.5 eV (Figure 1) show that the parent ion of the precursor (*m*/*z* 174) is not stable and extensive dissociative ionization occurs at room temperature, giving rise to peaks at *m*/*z* 57, 59, 112, 115, and 119. At ca. 760 K a small peak is seen at *m*/*z* 62, which is assigned to carbonic acid **1**. Isobutene (C_4_H_6_, *m*/*z* 56) is formed as a byproduct of the reaction in Scheme 2. Lower mass products, such as *m*/*z* 28 and 41, are likely produced via parallel decomposition channels of **2** or dissociative photoionization of, for example, isobutene.^48^ Increasing the pyrolysis temperature to above 900 K increases the conversion of precursor **2** but leads to full depletion of **1** producing mainly water and CO_2_, emphasizing its reactive character. Further mass spectra of **1** at different conditions along with ion velocity map imaging (VMI) of *m*/*z* 62 (Figure S1) are detailed in the Supporting Information (SI) providing further evidence that **1** is indeed formed via pyrolysis of **2.**

**Figure 1:**
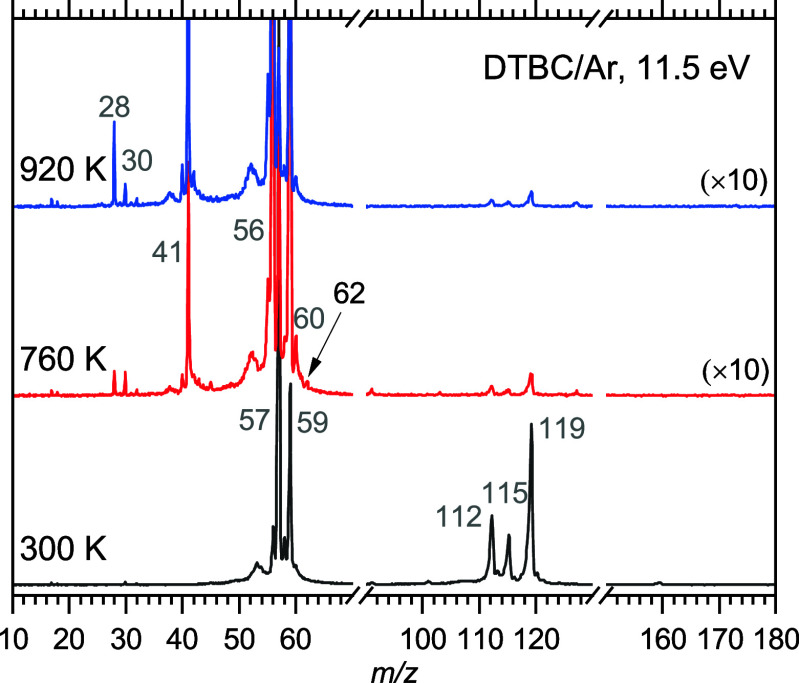
Mass spectra at 11.5 eV during the production of gaseous carbonic acid (H_2_CO_3_, **1**) from DTBC, **2**. Extensive fragmentation at 300 K is responsible for the formation of *m*/*z* 119, 115, 112, 59, and 57. **1** (*m*/*z* 62) and isobutene (*m*/*z* 56) are produced in parallel according to Scheme 2, while **1** rapidly decomposes above 760 K.

**Scheme 2:**
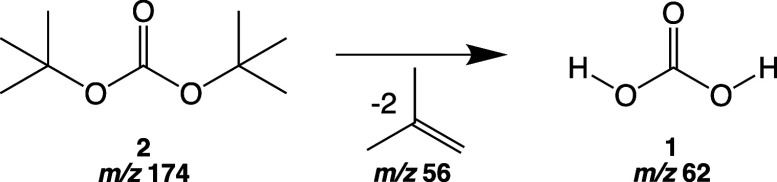
Decomposition of Carbonic Acid **1** via a Twofold Isobutene Loss from **2**(23)

The ms-TPES as well as photoionization (PI) spectra of carbonic acid **1**, formed at a reactor temperature of 790 K, are shown in Figure 2. Thanks to velocity map imaging (VMI) the 300 K room-temperature spectra can be obtained after rethermalization in the detection chamber, as detailed in the Supporting Information (Figure S2a,b). Moreover, the spectra do not have any contributions from neighboring masses, as shown in Figures S2c. The PI spectrum of *m*/*z* 62 starts to rise at around 11.18 eV and plateaus at 11.7 eV. On the one hand, the PI spectrum of carbonic acid is likely unique among *m*/*z* 62 species, but being broad and featureless, it lacks conformer-selectivity. The ms-TPES of *m*/*z* 62, on the other hand, clearly shows four intense peaks at 11.27, 11.45, 11.64, and 11.81 eV. Additional low-intensity bands are detected at 11.39, 11.50, 11.59, and 11.70 eV. We calculated adiabatic ionization energies (AIEs) of the three conformers, **1cc**, **1ct**, and **1tt**, using composite and EOM-CCSD methods as shown in Table 1 and Table S1, and found them to be 11.29, 11.22, and 11.08 eV, respectively, at the W1BD level of theory, which usually delivers better than chemical accuracy (1 kcal mol^–1^, 43 meV, 4.2 kJ mol^–1^).^49^ According to the calculated potential energy surface of **1**,^22,23^**1tt** is 42 kJ mol^–1^ less stable than **1cc** and **1ct** with a low-energy transition state in between. In thermal equilibrium at a reactor temperature of 790 K, the relative abundance of **1cc**:**1ct**:**1tt** is predicted to be 2.6:1:4.4 × 10^–3^ at the W1BD level of theory, meaning that **1tt** is unlikely to contribute to the signal above the detection limit. Thus, based on the ms-TPE and PI spectra, the first intense peak at 11.27 eV in the former and the onset of the latter at 11.18 eV are assigned to the AIEs of **1cc** and **1ct**, respectively, with the help of Franck–Condon (FC) simulations (see below). These numbers are also in good agreement with the W1BD calculations, with a difference of 20 and 40 meV for **1cc** and **1ct**, respectively.

**Figure 2:**
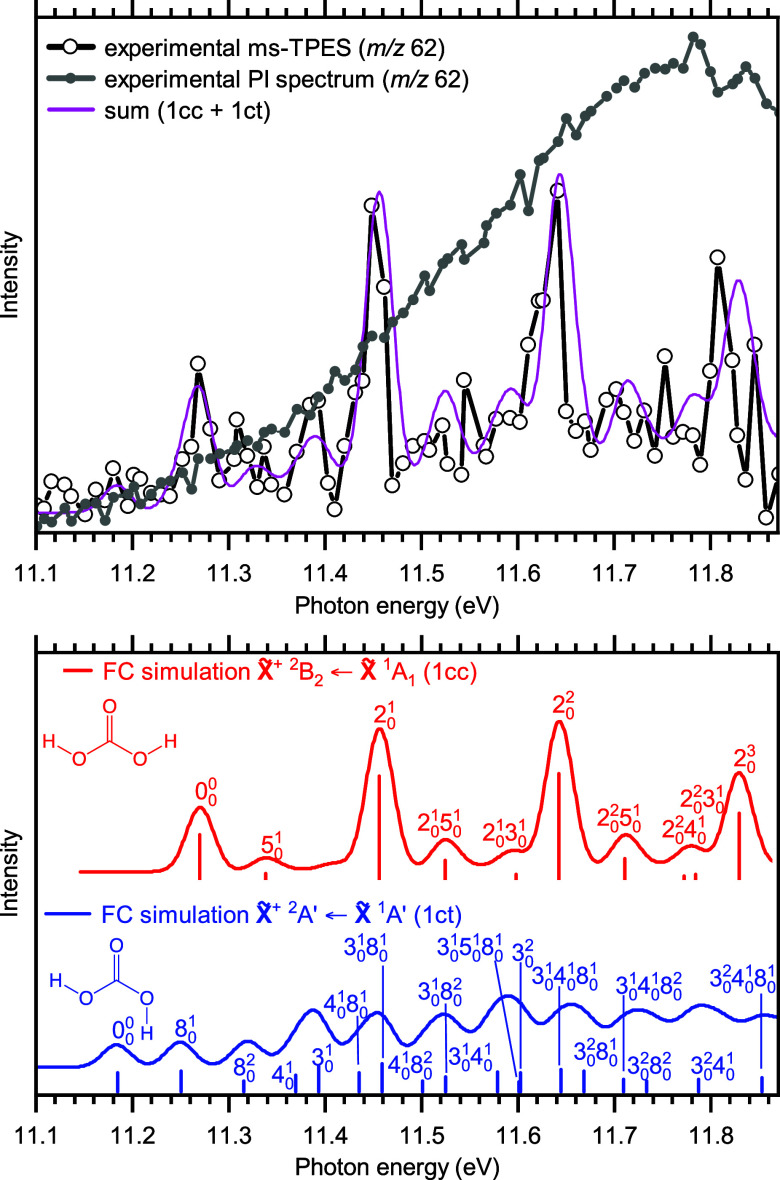
Photoion mass-selected threshold photoelectron (ms-TPE) and photoionization (PI) spectra of carbonic acid **1** (black and gray). Colored lines and sticks are Franck–Condon simulations for the transition from neutral to cation ground states of **1cc** (red) and **1ct** (blue) calculated at 300 K and at the B3LYP/6-311++G(d,p) level of theory. The stick spectra are convolved with a Gaussian function with a full width at half-maximum of 33 meV to account for the rotational envelope. The sum of the **1cc** and **1ct** simulations is colored magenta and represents the features in the experimental spectrum well.

**Table 1:** Experimental and Calculated Adiabatic Ionization Energies (AIEs) of the Three Conformers of Carbonic Acid **1**

AIE (eV)	**1cc**	**1ct**	**1tt**
Experiment	11.27 ± 0.02	11.18 ± 0.03^a^	
G4	11.23	11.16	11.02
CBS-APNO	11.27	11.21	11.08
W1BD	11.29	11.22	11.08
EOM-IP-CCSD/cc-pVQZ	11.22	11.16	

a Determined based on the FC simulation fitted to the ms-TPES.

To assign the conformers of **1** and to obtain spectroscopic insights, Franck–Condon (FC) simulations were fitted to the bands of the ms-TPES (Figure 2). The simulation of **1cc** (red lines, Figure 2, Figure S3b) is in good agreement with the intense peaks at 11.27, 11.45, 11.64, and 11.81 eV. Thus, the first clear peak at 11.27 ± 0.02 eV is assigned to the AIE of **1cc**, i.e., the transition into the cations’ (**1cc^+^**) vibrational ground state, also in excellent agreement with the composite method results (Table 1). The following intense peaks (11.45, 11.64, and 11.81 eV) are assigned to a C=O/C–OH stretching mode of **1cc^+^** (ν_2_^+^) with a progression of ca. 183 ± 20 meV (1478 ± 160 cm^–1^, see Figure 2), which compares well with the calculated value of 1503 cm^–1^ at the B3LYP/6-311++G(d,p) level of theory (Table S2 in SI). Exciting an HO–C–OH bending mode, ν_5_^+^, and combination bands of ν_5_^+^ and (ν_2_^+^) give rise to the low-intensity bands in the ms-TPES at 11.34, 11.52, and 11.71 eV, respectively.

However, the FC spectrum of **1cc** does not explain the bands at 11.39 and between 11.55 and 11.65 eV, as shown in Figure S3a,b in the Supporting Information. This could be remedied by adding the FC simulation of **1ct** (blue lines, Figure 2), which, unlike the FC simulation of **1cc**, shows a long progression with only a few well-resolved peaks. We relied on the most intense of the remaining bands at 11.39 eV and between 11.55 and 11.65 eV to reproduce the missing bands in the ms-TPES using the **1ct** FC simulation (Figure 2 and Figure S3c). This resulted in a good match to all observed features of the experimental spectrum (magenta lines in Figure 2). Generally, FC factors for **1ct** are only one-third of those for **1cc**, which makes a quantitative analysis of the relative abundances challenging. Furthermore, autoionization may contribute to the threshold ionization signal of the conformers differently, and the photoelectron dipole matrix elements may also be different for the conformers; therefore, a discussion of the relative ratio of **1cc** and **1ct** may be misleading. Alternative fits, where the 0–0 transition of **1ct** was set to 11.21 and 11.24 eV, respectively, are discussed in the SI (Figure S4a,b) and were disregarded. Based on the fitted **1ct** FC spectrum and the rising edge of the PI spectrum, the experimental AIE of **1ct** is determined to be 11.18 ± 0.03 eV, with a slightly larger uncertainty than for **1cc**. This is found to be well within single and averaged composite method calculations (see Table 1 and Table S1), with expected chemical accuracy (1 kcal mol^–1^/4.2 kcal mol^–1^/43 meV).^49^ Major vibrational modes of **1ct**^**+**^ are an HO–C–OH bending mode (ν_8_^+^) and two C=O/C–OH stretching vibrations (ν_4_^+^ and ν_3_^+^), shown in Figure 2. To test the sturdiness of the assignment, we also conducted FC simulations using different levels of theory and found that they were generally consistent with the B3LYP results, which agreed best with the experimental ms-TPES (Figure S5). Thanks to the good overlap of the simulation and the experimental spectra, two conformers of carbonic acid **1**, **1cc** and **1ct**, can be identified as the spectral carriers of the ms-TPES. Beyond the sensitive detection of **1** in reactive mixtures, the spectra also allow for the assignment of two conformers out of the three possible. The much lower computed ionization energy of the high-energy **1tt** conformer implies that its photoionization signal could likely be assigned selectively too, if abundant enough. Thus, the combination of photoelectron and photoionization spectroscopy is a well-suited tool for the conformer-selective identification of carbonic acid in photoionization-based experiments to unveil the chemistry of the ISM in terrestrial experiments. As the heat of formation of neutral **1** is listed in the Active Thermochemical Tables (ATcT),^50^ the experimental ionization energies can be simply added to derive the enthalpies of formation of the cation conformers as Δ_f_*H*°_0K_ = 485 ± 2 and 482 ± 3 kJ mol^–1^ for **1cc^+^** and **1ct**^**+**^, respectively. These thermochemical parameters can be utilized to calculate reaction enthalpies in ion–molecule reactions leading to or from **1**^**+**^ in astrochemical models.^51,52^

Besides acting as a fingerprint for the detection of **1** in reactive mixtures, the ms-TPES provides insights into the geometry change upon ionization and, thus, into the electronic structure of **1cc** and **1ct**. The highest occupied molecular orbitals (HOMOs) of **1cc** and **1ct** are shown in Figure 3. Both show nonbonding character at the oxygen atoms. The bare oxygen atom (O2) possesses the largest contribution, while the other two oxygen atoms (O1 and O3) have only smaller ones. Upon ionization of **1cc**, the O1–C–O3 and two H–O–C angles increase (Table S3 in the SI) due to less repulsion from the lone pairs by reducing the electron density on the oxygen atom. Consequently, the C=O bond length elongates, while two C–OH bonds shorten, resulting in three almost equal C–O bond distances upon ionization (Table S3). It is intriguing to mention that the intramolecular O···HO distance also increases upon ionization in **1cc** (2.32 to 2.39 Å) and **1ct** (2.17 to 2.37 Å), respectively. This may be explained by the increased positive polarization at the oxygen atoms upon ionization, which may lower their hydrogen bond acceptor capability in the cation. Spectroscopically, this change in geometry is mirrored by two totally symmetric vibrational modes of **1cc^+^** observed in the ms-TPES, the HO–C–OH bending (ν_5_^+^, a_1_) and the C=O/C–OH stretching (ν_2_^+^, a_1_) vibration. A similar geometry change occurs also in **1ct**, but due to its lower symmetry (C_s_ vs C_2v_), two vibrational modes are active, differing in the *cis* and *trans* C–OH stretch contributions (ν_4_^+^ = 1492 cm^–1^ and ν_3_^+^= 1685 cm^–1^ both a′). The equivalent mode (ν_10_^+^) in **1cc** is not active, due to its b_2_ symmetry. This intensity sharing in **1ct** leads to smaller FC factors as compared to those in **1cc**. However, the photoelectron spectra of **1cc** and **1ct**, in which the peaks for **1ct** are less intense than those of **1cc**, were successfully obtained using PEPICO spectroscopy. It is also worth mentioning that **1ct**^**+**^ and **1cc^+^** are quasi-isoenergetic at the W1BD level of theory or by utilizing the experimentally determined AIEs together with the Δ_f_*H*°_0K_ of the neutral. This may suggest a close-to-equal **1ct**^**+**^ and **1cc^+^** abundance in equilibrium when the cation is formed in the interstellar medium.

**Figure 3:**
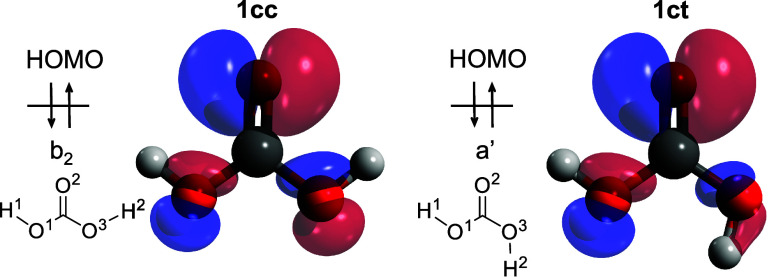
Highest occupied molecular orbitals (HOMO) of the two conformers of carbonic acid **1**, (left) **1cc**, and (right) **1ct**. The numbering of each atom in the discussion is also presented.

In conclusion, carbonic acid (H_2_CO_3_, **1**) was produced by flash pyrolysis of di-*tert*-butyl carbonate (**2**)^23^ and detected utilizing photoelectron photoion coincidence (PEPICO) spectroscopy with vacuum ultraviolet (VUV) synchrotron radiation. Based on the recorded PI spectrum and ms-TPES and Franck–Condon simulations, the two most stable conformers of **1**, *cis–cis* (**1cc**) and *cis–trans* (**1ct**), could be identified. Together with the adiabatic ionization energies (AIEs), these conformer-specific spectroscopic fingerprints are accessible in the PhotoElectron PhotoIon Spectral COmpendium (PEPISCO) database.^53^ Our spectroscopic data lay the foundation for employing photoionization and photoelectron spectroscopic methods to identify and ideally quantify **1cc** and **1ct** in terrestrial photoionization experiments to study astrochemically relevant reactions at VUV synchrotron facilities around the globe^31,40,41,54^ to probe the gas-phase formation of **1**, via, for example, radical recombination reactions (HOCO + OH) similar to surface pathways.^9^ In addition, our measured PI and TPE spectra and the determined AIEs may help to enable time-resolved pump–probe photoionization experiments from nano- down to femtoseconds to measure the lifetime and the fate of excited **1**, which may contribute to improve astrochemical models. Furthermore, our ms-TPES and computational analyses provide thermochemical parameters, such as ionization energies and heats of formation, as well as insights into the electronic structure of **1**, especially regarding molecular orbitals and geometries of both the neutral and cation.

## Experimental Section

The experiment was conducted utilizing the double imaging photoelectron photoion coincidence (*i*^2^PEPICO) endstation at the vacuum ultraviolet (VUV) beamline of the Swiss Light Source (SLS) located at Paul Scherrer Institute, Switzerland.^54−57^ Di-*tert*-butyl carbonate purchased from abcr GmbH (95% purity) was used as a precursor to produce carbonic acid (H_2_CO_3_, **1**) through flash vacuum pyrolysis. The precursor was placed in a container at a pressure of ca. 150 mbar at room temperature between a mass flow controller (MKS Instruments Inc.) and a high-vacuum molecular beam (MB) source chamber (10^–5^ mbar). The vaporized precursor was introduced into a resistively heated Chen-type SiC microtubular reactor^58^ (ca. 40 mm length, 1 mm inner diameter, 2 mm outer diameter, and 15 mm heated length) installed in the MB source chamber at a flow rate of 20 sccm argon (≥99.998%, PanGas) or helium (≥99.996%, PanGas). The correlation between the reactor temperature and output wattage had been calibrated before. The gas temperature close to the reactor centerline is predicted to be approximately 10% lower than the reactor surface temperature.^59,60^ Pressure and residence time in the reactor are estimated to be around 10–20 mbar and 10–50 μs, respectively.^58−60^ The pyrolyzed gases leaving the reactor expand into a high vacuum (10^–5^ mbar), forming a MB. The MB is skimmed using a Model 2 nickel skimmer (Beam Dynamics Inc., 2 mm aperture) and enters the ionization chamber (10^–7^ mbar) of the PEPICO spectrometer, where it is photoionized by VUV synchrotron radiation. VUV light is provided by a bending magnet, collimated onto a plane blazed grating (150 grooves mm^–1^) with a resolving power of 1500, and focused on the exit slit (200 μm). A differentially pumped rare gas filter between the focusing mirror and the endstation, filled with a neon/argon mixture at 8.5 mbar over 10 cm suppresses higher-order radiation from the grating.^55^ Photoions and photoelectrons are accelerated in opposite directions under a constant electric field of 218 V cm^–1^ and detected by position-sensitive delay line anode detectors (DLD40, Roentdek) in delayed coincidence.^61^ This enables time-of-flight (TOF) detection for cations and velocity map imaging (VMI) of both cations and electrons. Photoion mass-selected photoelectron spectrum (ms-TPES) as well as photoionization (PI) spectrum were recorded by scanning the photon energy in 10 meV steps from 11.10 to 11.87 eV with an integration time of 350 s per point. As for the ms-TPES, electrons with less than 10 meV kinetic energy were selected based on the photoelectron VMI. The threshold electrons in coincidence with cations in the room-temperature background signals arriving in the TOF range of interest were extracted based on the ion VMI to suppress hot band contributions.^62^ Contributions of hot electrons that possess higher kinetic energy without off-axis momentum components were also subtracted.^63^

Gaussian 16^64^ and Q-Chem 4.3^65^ were used for the computations. Geometry optimization and vibrational frequency calculations of the ground states were performed at the B3LYP/6-311++G(d,p), ωB97X-D/6-311++G(d,p), M06/6-311++G(d,p), G3, G4, CBS-QB3, CBS-APNO, W1BD, MP2/6-311++G(d,p), and CCSD/cc-pVTZ levels of theory. We used the Mulliken notation for the numbering of unscaled vibrational modes. Cation excited-state calculations were performed at the TD-B3LYP/6-311++G(d,p) level of theory but will contribute to the ms-TPES only above the investigated energy range. Adiabatic ionization energies were calculated with the aforementioned methods as well as at the (EOM-IP-)CCSD/cc-pVQZ level of theory, while the geometry optimization and vibrational frequencies were calculated utilizing (EOM-IP-)CCSD/cc-pVDZ. Franck–Condon (FC) simulations were performed at 300 K with the Franck–Condon–Herzberg–Teller method implemented in Gaussian 16,^64^ and the stick spectra were convolved with a Gaussian function with a full width at half-maximum of 33 meV.

## Supporting Information Available

Figure S1: Additional mass spectra and velocity map images; Figures S2, S3 and S4, S5: obtained room temperature ms-TPES (*m*/*z* 59, 60, 62) and additional Franck–Condon fits; Tables S1–S3: Calculated ionization energies, optimized geometries, and vibrational frequencies (PDF)
