# Supplementary material for: Conformer-Specific Photoelectron Spectroscopy of Carbonic Acid: H2CO3
Source: J Phys Chem Lett. 2024 Mar 1;15(10):2658–64. doi: 10.1021/acs.jpclett.4c00343 (PMC10945571; doi:10.1021/acs.jpclett.4c00343)
Supplement: Supplementary file 1 — jz4c00343_si_001.pdf [file jz4c00343_si_001.pdf]

Supporting Information

Conformer-Specific Photoelectron Spectroscopy of Carbonic Acid: H_2CO_3

Keisuke Kanayama,[a,b,c] *Hisashi Nakamura,*[b] *Kaoru Maruta,*[b] *Andras Bodi,*[a]

*Patrick Hemberger**[a]

[a] Laboratory for Synchrotron Radiation and Femtochemistry Paul Scherrer Institute

CH-5232 Villigen PSI (Switzerland)

[b] Institute of Fluid Science Tohoku University 2-1-1 Katahira, Aoba, Sendai, Miyagi 980-8577 (Japan)

[c] Graduate School of Engineering Tohoku University 6-6 Aramaki Aza Aoba, Aoba, Sendai, Miyagi 980-8579 (Japan)

Corresponding Author

* Patrick Hemberger, patrick.hemberger@psi.ch

1/ H_2CO_3 production

We attempted to produce H_2CO_3 (**1**) by heating of NH_4HCO_3 [25] and di-*tert*-butyl carbonate (**2**) [23] as precursors. In addition to **2**, diethyl carbonate (**3**) was also used as a precursor to produce **1** via twofold ethylene (C_2H_4) loss as suggested by Bucher et al.[44] However, **1** was not sufficiently produced either with the method using NH_4HCO_3, similarly to the results of Reisenauer et al.,[23] or by the flash pyrolysis of **3**, the latter being likely due to a competitive and more preferable reaction channel producing CO_2 and ethanol over a barrier 20–25 kJ mol^{-1} below that of **1** formation.[44,45] The results shown in the present study are therefore based on flash pyrolysis of **2**.

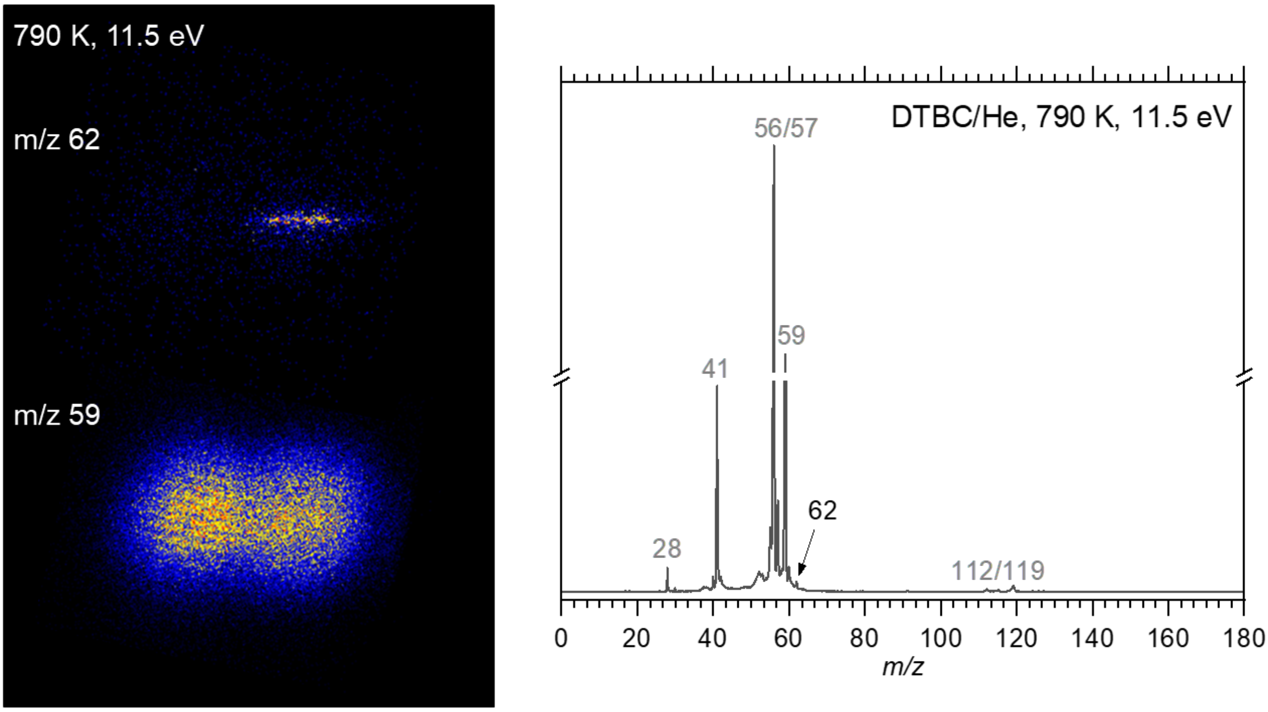

Figure S1. (left) Ion velocity map images (VMI) of *m/z* 62 and 59, which indicate that the *m/z* 62 signal results from direct photoionization of pyrolysis products and is not affected by dissociative photoionization (DPI) signal of the precursor **2** or any other pyrolysis intermediate. The VMI of *m/z* 59, on the other hand, shows broadening perpendicular to the MB axis, indicative of kinetic energy release in the dissociative ionization of the precursor **2**. (right) Mass spectrum of di-*tert*-butyl carbonate (**2**) pyrolysis at 790 K diluted with helium at photon energy of 11.5 eV.

2/ Contributions of the molecular beam, room temperature background and neighboring mass (*m/z* 59 and 60) signals to the ms-TPES

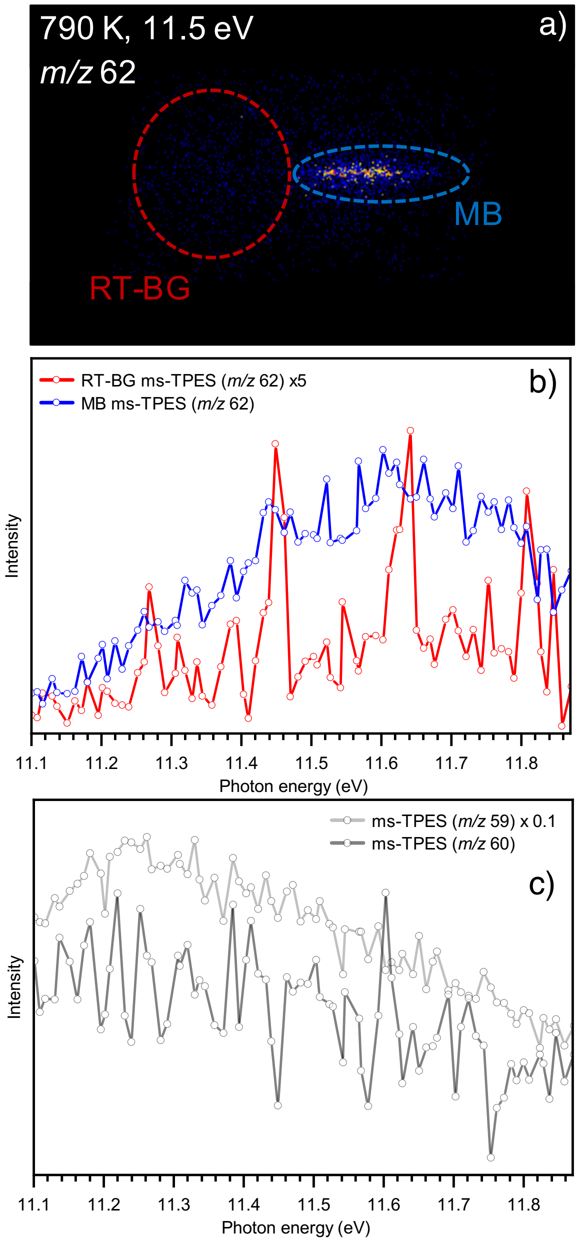

Figure S2. a) The 11.5 eV ion velocity map image (VMI), taken at a reactor temperature of 790 K, shows molecular beam (MB) as well as room temperature background (RT-BG) ions. The ms-TPES of the photoions at *m/z* 62 (b), obtained by integrating the MB (blue lines, lower trace), shows a broad and unstructured band, indicative for extensive hot- and sequence band transitions and a rovibrational temperature close to the one of the reactor.[62] The room temperature (RT-BG, red lines b)), on the other hand, shows a clearly pronounced vibrational structure, as the molecules collide with the ion optics and chamber wall to efficiently rethermalize after only a few collisions to a room temperature internal energy distribution. The neutrals diffuse back into the ionization region and contribute to the RT-BG component. This leads to less hot and sequence band transitions being excited upon photoionization and to well resolved peaks in the ms-TPES, justifying a Franck–Condon simulation at 300 K. c) ms-TPES of the dissociative ionization products at *m/z* 59 and 60, taken under the same conditions, do not show any overlapping features with the spectrum of m/z 62.

3/ Franck–Condon simulations

Different levels of theory and ms-TPES assignments were carried out to see the feasibility of the present FC simulation results.

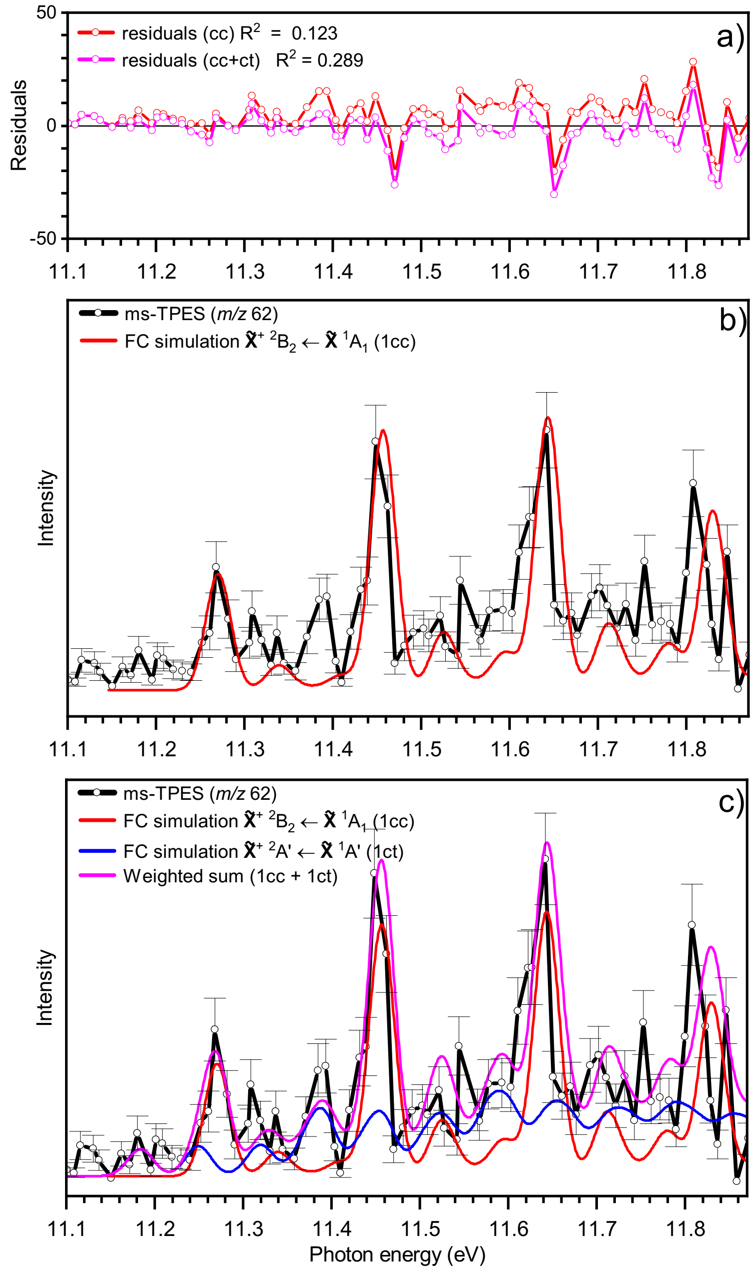

Figure S3. Residuals of the **1cc** and **1cc+1ct** fit (a). The comparison of the FC simulation (300 K) of **1cc** with the experimental spectrum shows that the bands at 11.39 and 11.55–11.64 eV are underestimated (a and b). The addition of the **1ct** simulation (c) leads to a better agreement of the model with the experimental spectrum (R^2 0.123 vs. 0.289 / lower residuals a). Especially the peak at 11.39 eV is much better represented by the model, which is also closer to the uncertainty bars of the experiment. Experimental error bars are defined as $\pm\sqrt[2]{N}$.

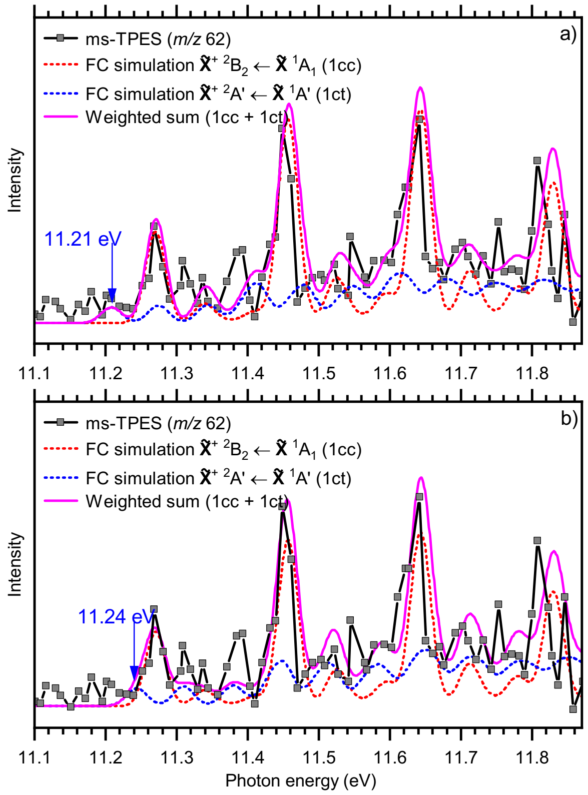

Figure S4. To further test the sturdiness of our fit, the 0-0 transition of the **1ct** FC simulation was set to 11.21 eV (a) and 11.24 eV (b), respectively. Together with the fit at 11.18 eV (Figure S3c), the three simulations span a range of 11.21 ± 0.03 eV around the composite method calculations (Table S1). From the three simulations the ones at 11.21 and 11.24 eV led to inferior fits, providing further evidence that the AIE of **1ct** is 11.18 eV.

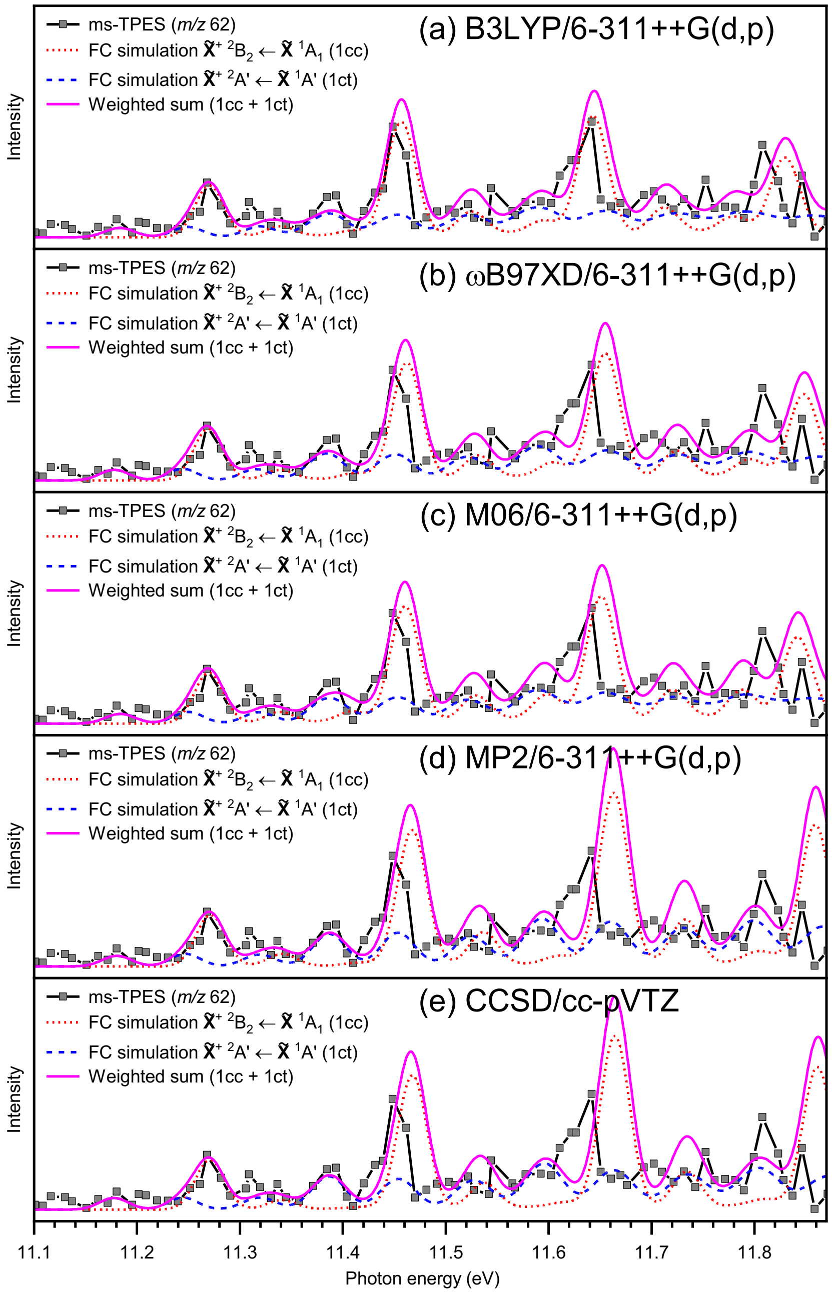

Figure S5. Franck–Condon (FC) simulations at different levels of theory. The weights of **1cc** and **1ct** in the weighted sum lines (magenta) are fixed in all cases. The B3LYP result (a) shows generally good agreement with the ms-TPES. The MP2 (d) and CCSD (e) results reproduce the band at 11.39 eV well but overestimate the intensity of the main peaks, e.g., the peak at around 11.64 eV in the ms-TPES. The slight blue-shift in the FC simulation at the higher photon energies, e.g., the peak at around 11.81 eV, is due to anharmonicity at high vibrational excitation being disregarded in the double harmonic approximation.

Table S1. Calculated adiabatic ionization energies (AIEs) of *cis-cis* (**1cc**), *cis-trans* (**1ct**), and *trans-trans* (**1tt**) carbonic acid (**1**) at different levels of theory.

Method / AIE (eV)	**1cc** (C_{2v})		**1ct** (C_s)		**1tt** (C_2)	
	$\tilde{X}^{+\,2}B_2$	$\tilde{A}^{+\,2}B_1$	$\tilde{X}^{+\,2}A'$	$\tilde{A}^{+\,2}A''$	$\tilde{X}^{+\,2}B$	$\tilde{A}^{+\,2}B$
ms-TPES	11.27		11.18		-	
(TD-)B3LYP/6-311++G(d,p)	11.16	11.94	11.11	11.88	10.98	11.78
ωB97XD/6-311++G(d,p)	11.17		11.09			
M06/6-311++G(d,p)	11.27		11.21			
G3	11.28		11.22		11.08	
G4	11.23		11.16		11.02	
CBS-QB3	11.33		11.27		11.13	
CBS-APNO	11.27		11.21		11.08	
W1BD	11.29		11.22		11.08	
average CBS-APNO/G4	11.25		11.19			
average CBS-APNO/G3	11.28		11.22			
average G3/G4	11.26		11.19			
MP2/6-311++G(d,p)	11.36		11.24			
CCSD/cc-pVTZ	10.96		10.86			
EOM-IP-CCSD/cc-pVQZ	11.22		11.16			

Table S2. Unscaled vibrational frequencies in cm^{-1} of neutrals, cations of both **1cc** and **1ct** conformers at the B3LYP/6-311++G(d,p) level of theory. Mulliken numbering of the vibrational modes is given in parentheses. Franck-Condon active modes are highlighted in boldface.

Modes / (cm^{-1})	**1cc** ($\tilde{X}\ ^1A_1$)	**1cc⁺** ($\tilde{X}^{+\,2}B_2$)	**1ct** ($\tilde{X}\ ^1A'$)	**1ct⁺** ($\tilde{X}^{+\,2}A'$)
	519 (a_2, ν_6)	363 (a_2, ν_6^+)	471 (a'', ν_{12})	375 (a'', ν_{12}^+)
	550 (a_1, ν_5)	427 (b_2, ν_{12}^+)	543 (a', ν_9)	465 (a', ν_9^+)
	594 (b_1, ν_8)	537 (b_1, ν_8^+)	552 (a'', ν_{11})	504 (a'', ν_{11}^+)
	604 (b_2, ν_{12})	**550 (a_1, ν_5^+)**	608 (a', ν_8)	**529 (a', ν_8^+)**
	797 (b_1, ν_7)	745 (b_1, ν_7^+)	783 (a'', ν_{10})	746 (a'', ν_{10}^+)
	978 (a_1, ν_4)	**1049 (a_1, ν_4^+)**	963 (a', ν_7)	1041 (a', ν_7^+)
	1158 (b_2, ν_{11})	1122 (b_2, ν_{11}^+)	1143 (a', ν_6)	1113 (a', ν_6^+)
	1293 (a_1, ν_3)	**1143 (a_1, ν_3^+)**	1265 (a', ν_5)	**1140 (a', ν_5^+)**
	1449 (b_2, ν_{10})	**1503 (a_1, ν_2^+)**	1390 (a', ν_4)	**1492 (a', ν_4^+)**
	1826 (a_1, ν_2)	1543 (b_2, ν_{10}^+)	1881 (a', ν_3)	**1685 (a', ν_3^+)**
	3809 (b_2, ν_9)	3648 (b_2, ν_9^+)	3799 (a', ν_2)	3632 (a', ν_2^+)
	3811 (a_1, ν_1)	3660 (a_1, ν_1^+)	3802 (a', ν_1)	3646 (a', ν_1^+)

Table S3. Bond lengths and angles of **1cc** and **1ct** calculated at the B3LYP/6-311++G(d,p) level of theory. See **Figure 3** in the manuscript for the atom numbering.

Bond length (Å)	**1cc** ($\tilde{\mathbf{X}}\ ^1A_1$)	**1cc⁺** ($\tilde{\mathbf{X}}^{+\ 2}B_2$)	**1ct** ($\tilde{\mathbf{X}}\ ^1A'$)	**1ct⁺** ($\tilde{\mathbf{X}}^{+\ 2}A'$)
O1–H1	0.966	0.979	0.967	0.980
C–O1	1.340	1.279	1.360	1.287
C=O2	1.205	1.277	1.195	1.286
C–O3	1.340	1.279	1.339	1.268
O3–H2	0.966	0.979	0.966	0.979
Bond angle (°)	**1cc** ($\tilde{\mathbf{X}}\ ^1A_1$)	**1cc⁺** ($\tilde{\mathbf{X}}^{+\ 2}B_2$)	**1ct** ($\tilde{\mathbf{X}}\ ^1A'$)	**1ct⁺** ($\tilde{\mathbf{X}}^{+\ 2}A'$)
H1–O1–C	106.9	116.4	107.5	117.0
O1–C–O2	125.7	121.5	125.2	119.3
O1–C–O3	108.7	117.0	110.5	122.5
O2–C–O3	125.7	121.5	124.3	118.2
C–O3–H2	106.9	116.4	109.7	114.5

References

(23) Reisenauer, H. P.; Wagner, J. P.; Schreiner, P. R. Gas-Phase Preparation of Carbonic Acid and Its Monomethyl Ester. *Angew. Chem. Int. Ed.* **2014,** *53* (44), 11766-11771.

(25) Terlouw, J. K.; Lebrilla, C. B.; Schwarz, H. Thermolysis of NH4HCO3—A Simple Route to the Formation of Free Carbonic Acid(H2CO3) in the Gas Phase. *Angew. Chem. Int. Ed.* **1987,** *26* (4), 354-355.

(46) Bucher, G. Ester Pyrolysis of Carbonates: Bis(benzene hydrate) Carbonate as Potential Precursor for Monomeric Carbonic Acid. *Eur. J. Org. Chem.* **2010,** *2010* (6), 1070-1075.

(47) Sun, W.; Huang, C.; Tao, T.; Zhang, F.; Li, W.; Hansen, N.; Yang, B. Exploring the high-temperature kinetics of diethyl carbonate (DEC) under pyrolysis and flame conditions. *Combust. Flame* **2017,** *181*, 71-81.

(62) Hemberger, P.; Wu, X.; Pan, Z.; Bodi, A. Continuous Pyrolysis Microreactors: Hot Sources with Little Cooling? New Insights Utilizing Cation Velocity Map Imaging and Threshold Photoelectron Spectroscopy. *J. Phys. Chem. A* **2022,** *126* (14), 2196-2210.
